# An in vivo evaluation of anti-inflammatory, analgesic and anti-pyretic activities of newly synthesized 1, 2, 4 Triazole derivatives

**DOI:** 10.1186/s12906-021-03485-x

**Published:** 2021-12-31

**Authors:** Tabinda Azim, Muhammad Wasim, Muhammad Shoaib Akhtar, Irfan Akram

**Affiliations:** 1 Department of Pharmacy, Iqra University Islamabad Campus, Islamabad, Pakistan; 2 Department of Pharmacy, Abasyn University Islamabad Campus, Islamabad, Pakistan; 3 Faculty of Pharmacy, Department of Pharmacology, University of Sargodha, Sargodha, Pakistan; 4 Faculty of Pharmacy, Department of Pharmacology, Islamia University, Bahawalpur, Pakistan

**Keywords:** 1, 2, 4-Triazoles, Anti-inflammatory, Paw edema, Anti-pyretic, Anti-nociceptive, Post-operative pain

## Abstract

**Background:**

In recent years, 1, 2, 4-triazole and its derivatives have been reported to be pharmacologically significant scaffolds. They possess analgesic, anti-tubercular, anti-inflammatory, anti-convulsant, anti-oxidant, anti-fungal, anti-cancer, anxiolytic and anti-depressant activity. This study was designed and conducted to evaluate the potential anti-inflammatory, analgesic and antipyretic activities of Triazole derivatives.

**Methods:**

Swiss albino (male and female) mice weighing 20-30 g (10-24 weeks female), (5-14 weeks male) and Wister Kyoto rats (male and female) weighing 200-300 g (8-10 weeks old) were used for the present study. Anti-inflammatory activity was checked using Lambda carrageenan (λ) and egg albumin-induced paw edema models. Analgesic via Writhing Reflex induced by acetic acid and formalin, furthermore anti-pyretic activity was assessed by yeast induced pyrexia.

**Results:**

Both of the test compounds exhibited encouraging anti-inflammatory analgesic and antipyretic results when compared with standard drug ibuprofen. The maximum inhibition of edema for the compound (S)-1-(4-Amino-5-mercapto-4H-1,2,4-triazole-3-yl) ethanol *[3]* was found to be (91)% as compared to reference drug ibuprofen (82)%, while (S)-1-(6-Phenyl-7H-[1,2,4]triazolo[3,4-b][1,3,4]thiadiazin-3-yl)ethanol *[5e]* showed equipotent results to ibuprofen (81)%. The derivatives were also screened for their anti-nociceptive activity by Acetic acid writhing and tail immersion test. Compound 3 showed a significant reduction in wriths (83)% as compared to standard drug ibuprofen 71.5% and [5] showed comparable results to ibuprofen by exhibiting 70% reduction in writh at the same dose as that of standard drug, moreover, there were no signs of toxicity being observed after administration of high doses of test compounds to mice.

**Conclusions:**

It is evident from the results that compounds 3(compound A) and 5(compound B) are a potential candidate for anti-inflammatory, analgesic and anti-pyretic and the scaffold could be used for further structural modifications. Further studies would help to evaluate their molecular mechanism of action regarding these beneficial activities.

## Background

Inflammations have been reported to operate during severe perturbations of homeostasis, such as tissue injury, infections, exposure to contaminants. Thus considerable efforts have been made to understand the cellular and molecular events behind the acute inflammatory response to infection [1]. Inflammation is also believed to be a major culprit in arthritis, lupus, high blood pressure, migraines, rheumatoid arthritis, Chron’s disease, Alzheimer’s disease, irritable bowel syndrome, colitis, tendonitis etc. [2].

Pain is an unpleasant experience specifically evoked by tissue injury or potential, noxious (i.e., tissue-damaging) stimuli. Its protective mechanism causing individual awareness to withdraw the affected area from stimuli causing pain [3]. Pain transduction, transmission and perception are very extensive and complex interplays between signalling systems, modulation from higher centres, involving fundamental biological events at various levels of the nervous system and the unique perception of the individual [4].

Although pain is part of a defensive reaction against the dysfunction of an organ or imbalance in its functions against the potentially dangerous stimulus and is necessary for survival, persistent pain can cause anxiety, depression and a reduction in the quality of life. Postoperative pain is still a critical clinical problem, studies have shown that about 74% of the patients experience moderate to severe pain after being discharged from emergency wards. A high percentage of cancer chemotherapy patients report the inefficient management of pain they experience [5]. Current pain management includes analgesics like (NSAIDs) and opioids, having limited safety and effectiveness and are associated with multiple side effects including, respiratory depression, nausea, vomiting, liver failure and addiction. Further research is needed to develop more safe and effective derivatives for efficient pain management [6].

In vertebrates, fever is a complex adaptive mechanism against infections, triggered by aseptic stimuli. It involves a cytokine-mediated rise in core temperature, stimulation of endocrinologic and immunological systems. There is increased release of prostaglandin E_2_ (PGE_2_) within parts of the brain, leading to altered neuronal firing in the hypothalamus eventually rising body temperature. Fever is considered as body defense to establish a non-survivable environment for infectious agents [7].

Current drug management of inflammation pain and fever includes utilization of non-steroidal anti-inflammatory drugs (NSAIDs), slow-acting disease-modifying anti-rheumatic drugs (DMARDs), glucocorticoids, immunosuppressant and biologics that specifically target the inflammatory cytokines [8]. However, their potential toxicities like gastrointestinal problems, respiratory depression, renal damages and possible dependence (with opioids) are still enduring dilemma of the medical world. This scenario highlights the need and provides a basis for the search and evaluation of new natural or synthetic chemical compounds for the development of novel, safe, and effective anti-inflammatory, anti-pyretic and analgesic compounds [9, 10]. In the past years, the triazole derivatives have gained considerable attention due to their diverse pharmacological activities. They are of great importance to chemists especially, owing to their wide spectrum of activities, better pharmacokinetic and pharmacodynamics profile [11]. 1,2,4 triazoles and their fused heterocyclic derivatives are known to have a broad spectrum of therapeutic activities like neuro-protectant [12], antimalarial, PDE4A inhibitors [13],anti-tubercular, anxiolytic, anti-convulsant, anti-fungal, anti-oxidant, analgesic, antimicrobial [14], anti-inflammatory, anti-cancer [15, 16], anti-asthmatic, diuretic, anti-fungal [17], and anti-Alzheimer properties [18, 19]. 1,2,4-Triazoles are important pharmacophore under research focus due to their high affinity for biological receptors, dipole character, their hydrogen-bonding capacity, rigidity and solubility. The Triazole moiety is an integral part of the diverse range of drugs available these days including antifungal (voriconazole, itraconazole, fluconazole), hypnotic (alprazolam), anticancer (anastrozole), aromatase inhibitor (letrozole), muscle relaxant (etizolam), antiviral (ribavirin) and anticonvulsant (loreclezole) [20, 21].

Studies have also shown that 1,2,4 triazole derivatives having potent anti-inflammatory activity caused less gastrointestinal (GI) side effects than the reference drugs like naproxen and indomethacin did [22]. It has also been reported that derivatives of 1, 2, 4 triazole nucleus showed weaker antimicrobial activities than the derivatives that were fused with heterocyclic compounds like thiazole, thiazidine [23, 24].

Recent studies have elaborated the synthesis of new hydrazone derivatives of 1,2,4-triazole and evaluation of their diverse pharmacological activities both in vitro and in vivo. An anti-inflammatory study reported by Khan B. *et.al* *.* has revealed that the test compound 241a showed results comparable to the reference drug indomethacin. The binding mode and site of the compound to a key enzyme involved in inflammation, PTGS or COX was studied against ligand celecoxib. Docking studies showed that this compound attached to the celecoxib binding site in COX with high affinity [25].

In another study reported by Aliaa M. et al. 1,2,4-triazole/oxime hybrids (245a-c) were evaluated for their anti-inflammatory activity in rats. Results revealed significant anti-inflammatory activity of the test compounds in comparison to the reference drug indomethacin causing % age edema inhibition of 100, 101 and 111%, respectively [26]. Apart from anti-inflammatory activities, studies have been conducted to evaluate analgesic properties of 1,2,4 triazole derivatives. One such pharmacological screening of Schiff and Mannich bases derivatives of 1,2,4-triazoles conducted by Gowda et al. showed that compounds 247c and 247d showed satisfactory anti inflammatory activity in animal models compared to indomethacin while other compounds 247a, 247b and 247d showed significant analgesic effects [27].

Research has demonstrated that inhibition of p38α MAP kinase could be beneficial in treating chronic inflammatory diseases via inhibition of important cytokines including TNF-α and interleukins. Tariq S.et al has reported synthesis, anti-inflammatory activity and p38α MAP inhibition of new series of *N*-[3-(substituted-4*H*-1,2,4-triazol-4-yl)]-benzo[*d*]thiazol-2-amines (4a–n). In-vivo anti-inflammatory studies demonstrated, compound 4f to be most active (85.31%) when compared to standard drug diclofenac sodium(83.6%), with less ulcerogenic effects as well. Most importantly the p38α MAP kinase inhibition exhibited by this compound was superior to the standard SB 203580 used in this study [28]. In an effort to use the 1,2,4 Triazole scaffold to develop new analgesic anti-pyretic and anti-inflammatory candidates with less ulcerogenic effects, a lot of modifications are being implicated in the molecule. Derivatives synthesised by combining thiazolo[3,2-*b*]-1,2,4-triazole ring with ibuprofen, (*S*)-naproxen and flurbiprofen showed potential in-vivo and in vitro analgesic and anti-inflammatory activities without gastrointestinal side effect. Analgesic activity was assessed by Tail-flick test and anti-inflammatory by carrageenan-induced paw edema in mice [29].

Furthermore, the Pharmacological screening of 4-[{1-(aryl)methylidene}-amino]-3-(4-pyridyl)-5-mercapto-4H-1,2,4-triazole derivatives proclaim that compounds (3b-3d) exhibited significant analgesic activity comparable with the standard drug Analgin, in the tail-flick mice model, while compounds 3a, 3e, and 3f showed significant anti-pyretic activities compared with the standard drug aspirin in the yeast-induced pyrexia model [30]. Apart from analgesic and anti-inflammatory studies, the anti-pyretic activity of 1, 2, 4 triazole derivatives have been also assessed by the yeast method in rats, declared that all the synthesized compounds inherent antipyretic activity [31].

**Fig. 1:**
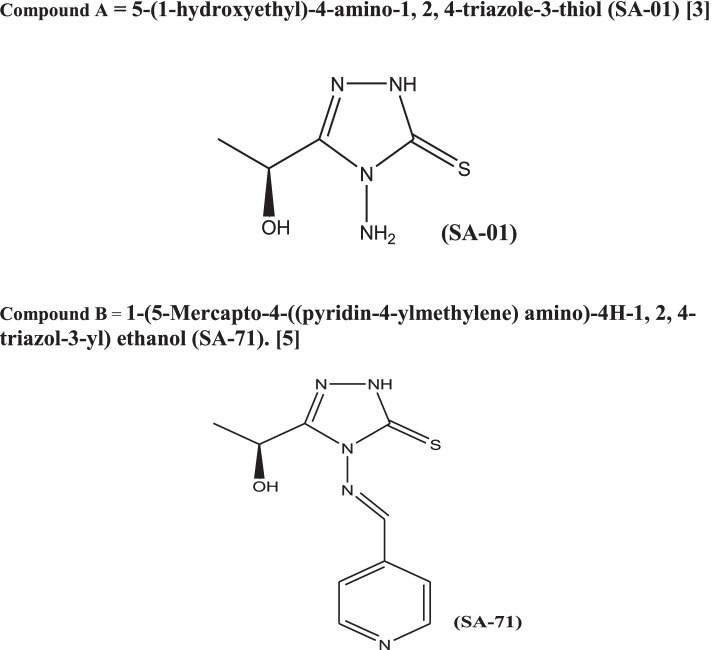
Compound **A** = 5-(1-hydroxyethyl)-4-amino-1, 2, 4-triazole-3-thiol (SA-01) [3]. Compound **B** = 1-(5-Mercapto-4-((pyridin-4-ylmethylene) amino)-4H-1, 2, 4-triazol-3-yl) ethanol (SA-71) [5]

All these studies provide us with encouraging evidence of significant anti-pyretic, analgesic and anti-inflammatory effects of 1,2,4 Triazole and enhance their importance as future candidates in multiple disease treatments. In the present study, we extended this ongoing research by conducting and reporting, in-vivo evaluation of anti-inflammatory, analgesic and antipyretic activities of newly synthesized derivatives of 1,2,4-triazole, namely compound A, (S)-1-(4-Amino-5-mercapto- 4H-1,2,4-triazol-3-yl)ethanol*[3]* and Compound B (S)-1-(6-Phenyl-7H-[1,2,4]triazolo[3,4-b][1,3,4]thiadiazin- 3-yl)ethanol*[5]* These new optically active compounds contain 1,2,4–triazole nucleus fused with a differently substituted 1,3,4-thiadiazine moiety, characterized by Mass, infrared (IR), Nuclear magnetic resonance (NMR) spectroscopy and single-crystal X-ray diffraction analysis. The anti-oxidant activity of all the synthesized compounds has been evaluated by DPPH radical scavenging [32] (Fig. 1).

## Methods

### Animals

*Swiss albino* (male and female) mice weighing 20-30 g (10-24 weeks female), (5-14 weeks male) and *Wister Kyoto* rats (male and female) weighing 200-300 g (8-10 weeks old) were used for the present study. Animals were given a week time to get acclimatized with laboratory conditions of the Department of Pharmacy, University of Sargodha, Sargodha, Pakistan. Animals were kept under standard laboratory conditions with a controlled environment of temperature 23 ± 3 C, humidity (60% ± 10%) an 12 h light/dark cycle. Animals were kept in polypropylene rat/mice cages in a group consisting of not more than six rats/mice per cage. They were given free access to food with a standard rodent pellet diet and drinking water. Animal handling was according to the National research guidelines [33]. Prior the animal experiment, our pharmacological protocols were approved by institutional ethical committee, College of Pharmacy, University of Sargodha, Pakistan with approval No (67B18 IAEC/UOS). All the animals (mice and rats) used in current study were released after completion of the specified studies.

### Drug and chemicals

Lambda carrageenan (λ) and dried brewer’s yeast were obtained from Sigma Chemical Company (St. Louis, MO, USA). Ibuprofen, was purchased from Global Pharmaceuticals (Islamabad, Pakistan) and DMSO from Spain (Barcelona, Spain). Formalin was acquired from Merck (MERCK, Darmstadt, Germany), Acetic acid from BDH Laboratories, (Poole, England). Test synthetic derivatives of 1,2,4-triazole, namely (S)-1-(4-Amino-5-mercapto-4H-1,2,4-triazol-3-yl)ethanol[3]and(S)-1-(6-Phenyl-7H [1,2,4]triazolo[3,4-b][1,3,4]thiadiazin-3-yl)ethanol[5] were synthesized by chemistry Department of Allama Iqbal Open University, Islamabad, Pakistan.

### Experimental design

#### Anti-inflammatory studies (carrageenan-induced paw edema in mice)

Acute inflammation was induced by injection of 0.1 ml of freshly prepared (λ) carrageenan solution (0.5% in distilled water) subcutaneously into the plantar surface of the right hind paw of the mice. Anti-inflammatory activities of newly synthesized compounds A and B were estimated by the model of acute inflammatory pain in mice using the carrageenan paw edema model reported by Heidari et al. [34] Swiss albino mice were fasted 24 **h** before the experiment with free access to water. All mice were divided into 8 groups, each group consisting of six mice of mix gender. Group, I served as the negative control group and received a vehicle (carrageenan+ DMSO in normal saline) dose of 10 ml/kg I.P [34]. Group II was administered Ibuprofen 100 mg/kg (i.p) as a reference standard drug [35]. Similarly, three different doses (25, 50 and 75 mg/kg for compound A while 50, 75 and 100 mg/kg for compound B) of the two synthesized compounds in DMSO were administered to the remaining six groups of animals 1 **h** before induction of inflammation.

Anterio-posterior diameter of the paw was measured at 0 h (before edematogenic agent injection) immediately after carrageenan injection and later at 1, 2 and 3 h intervals using digital vernier calliper at the marked site [36]. Edema was expressed as a mean increase in paw size (mm) with respect to the DMSO negative control group. Inhibition was expressed as a percentage increase or decrease in edematous paw size. The ability of the compounds to inhibit the foot edema was taken as an indication of an anti-inflammatory activity [37].

The following formula was used to determine the percentage % inhibition in edema.

$$\mathrm{Inhibition}\;\left(\%\right)=\frac{\left(\mathrm{Vt}-\mathrm{Vo}\right)^{\mathrm{control}}-\left(\mathrm{Vt}-\mathrm{Vo}\right)^{\mathrm{treated}}}{\left(\mathrm{Vt}-\mathrm{Vo}\right)^{\mathrm{control}}}\times100$$

Vt represents mice paw volume at time interval‘t’, Vo is the initial mice paw volume (basal value), (Vt- Vo) ^control^ is edema produced in the control group and (Vt- Vo) ^treated^ is edema produced in the treated group.

### Egg-albumin -induced paw edema

Anti-inflammatory activities of test compounds **A** and **B** were also evaluated using the egg albumin-induced paw edema according to the standard method reported [38]. Inflammation of the hind paw was induced by 0.1 mL of fresh egg white into the inner surface of the right hind paw of the mice. The diameter of the injected paw was measured before and immediately after injection. Measurement was done subsequently every hour over a period of 3 h. Edema was assessed as the difference between zero time diameter of the injected paw and diameter after administration of egg white during 3 h’ time period. Measurement was done using vernier callipers [39].

Group I was taken as a control group and was given DMSO (5%, 10 ml/kg, i.p). Group II was treated with the standard drug Ibuprofen (100 mg/kg, i.p). Similarly, three different test doses of the new compounds in DMSO (25, 50 and 75 mg/kg for compound A while 50, 75 and 100 mg/kg for compound B) were administered to the respective groups of mice. All the above treatments were given 1 h before induction of inflammation.

Anti-inflammatory effects of the drugs were calculated by the following equation:

$$\mathrm{Anti}-\mathrm{inflammatory}\ \mathrm{activity}\ \left(\%\right)=\left(\mathrm{C}-\mathrm{T}/\mathrm{C}\right)\ \mathrm{x}\ 100$$

Where T represent the percentage difference in paw volume after test compound administration and C the percentage difference in the volume of the control group [40].

#### Anti-nociceptive activity of the synthesized compounds

##### Acetic acid-induced writhing reflex in mice (chemical stimulus)

Analgesic activities of the compounds were evaluated by a standard reported method to induce acute pain [41]. Animals were fasted for 24 h before the experiment and were divided into eight groups having six mice each. Group I was given DMSO (10 ml/kg, i.p), group II was treated with standard drug Ibuprofen (100 mg/kg, i.p) and the rest of the groups were given three different doses of the test compounds (25, 50, 75 mg/kg for compound A while 50, 75 and 100 mg/kg for compound B) through IP route.

Thirty minutes post-treatment; mice in all groups were treated with 10 ml/kg of 3% acetic acid [42] v/v intraperitoneally. The mice were then left for 5 min, and the number of writhes was counted for the next 20 min. Writhe is defined as a contraction of the abdominal muscles accompanied by elongation of the body and extension of hind limbs [43]. The experiment was performed in a quiet laboratory with an ambient temperature of 22 ± 1 °C. The ability of the test compounds to significantly prevent the number of acetic acid-induced writhes were an indication of an anti-nociceptive activity [37].

Percentage inhibition of writhing was calculated using the following equation:

$$\%\mathrm{inhibition}=\frac{\mathrm{Mean}\;\mathrm{number}\;\mathrm{of}\;\mathrm{writhes}\;\left(\mathrm{control}\right)-\mathrm{Mean}\;\mathrm{number}\;\mathrm{of}\;\mathrm{writhes}\;\left(\mathrm{test}\right)}{\mathrm{Mean}\;\mathrm{number}\;\mathrm{of}\;\mathrm{white}\;\left(\mathrm{control}\right)}\times100$$

### Tail immersion test

The anti-nociceptive effect of the test substances was also evaluated using the hot tail immersion method described by Sewell and Spencer [44]. Mice were screened for sensitivity to hot water by the tail immersion method. The tails of mice were dipped gently into hot water maintained at 55 °C to 55.5 °C. Mice that were able to lift tail from hot water in 5 s were considered to be sensitive to heat and selected for the test. Whereas mice withstanding hot water for more than 5 s were excluded from the test, as the withdrawal time for the control mice usually lies between 1 to 5.5 s. A withdrawal time of more than 6 s was taken as a positive analgesic response. This test is specific for opioid-like central analgesic and helps to differentiate between central and peripheral analgesics [45].

Selected mice of either sex were divided into eight groups; each group was composed of six animals. Group I was given DMSO (10 ml/kg, i.p), Group II was treated with Ibuprofen (100 mg/kg, i.p), groups III-VIII were administered different doses of the test compounds (25, 50, 75 mg/kg for compound A while 50, 75 and 100 mg/kg for compound B) intraperitoneally. After 30 min of treatment, the tails of mice were marked up to 5 cm. The animal was kept in a vertical position to hang the tail, which was up to 5 cm into a pot of hot water maintained at 55 ± 0.5 °C. The time in seconds to withdraw the tail out of the water was taken as the reaction time (Ta). The reading was taken after 0, 15, 30 and 60 min time interval. The cut-off time, i.e. time of no response was put at 20 s to avoid damage to the tail, while Tb was considered the reaction time for the control group [46].

### Formalin-induced paw edema

The formalin test is a broadly used animal model of chemically induced persistent pain. Formalin injection into mice hind paw induces a spontaneous nociceptive behaviour like licking, flinching, or biting of the paw [47]. The method described by Tjølsen et al [48] was used to evaluate the analgesic effects of test compounds against formalin-induced nociception in mice. Following overnight fast mice were divided into eight groups with six mice each. Group, I was categorized as a control group receiving 10 ml/kg, i.p DMSO (5%).

Group II was treated with 100 mg/kg,i.p of ibuprofen. Group III-VIII were administered different doses of the test compounds (25, 50, 75 mg/kg for compound A while 50, 75 and 100 mg/kg for compound B) through IP route to the animals 60 min prior to the formalin injection. Twenty microliters (20 μl/0.02 ml) of 5% formalin in saline was injected subcutaneously into the right hind paw of each mice. The time (in seconds) spent on licking and biting of the injected paw was taken as a reaction to nociception and recorded in both the early phase (0–5 min) and late phase (15–30 min) after the formalin injection, representing the neurogenic and inflammatory pain responses, respectively. The time spent licking hind paw will be evaluated for analgesia expressed as percentage % inhibition of time spent licking (Fig. 3).

Following formula was used to determine % inhibition:

$$\%\mathrm{inhibition}=\frac{\mathrm{Licking}\;\mathrm{time}\left(\mathrm C\right)-\mathrm{licking}\;\mathrm{time}\;\left(\mathrm T\right)}{\mathrm{Licking}\;\mathrm{time}\;\left(\mathrm C\right)}\times100$$

Where C = control group, T = Test group.

#### Evaluation of anti-pyretic activity

##### Brewer’s yeast induced pyrexia in rats

Antipyretic activity of the test compounds was measured by the method described by Adams et al. [49] Wistar rats fasted overnight with water ad libitum before the commencement of the experiments. Pyrexia was induced by subcutaneously injecting 20% w/v brewer’s yeast suspension (10 ml/kg) into the animal’s dorsum region. Eighteen hours after the injection, the rectal temperature of each rat was measured using a thermometer. Rats that showed an increase in temperature of at least 0.7 °C were only subjected to experiments [7, 50]. After 18 h of yeast injection Group, I served as the control group was treated with DMSO 10 ml/kg I.P. Group II was administered the standard drug ibuprofen 100 mg/kg. In the same manner, three different doses of test compounds solution in DMSO were given by I.P route to all other groups of animals. Rectal temperature was noted at 1-h interval up to 4 h.

#### Acute toxicity and behavioural pattern studies

For acute toxicity method established by Kang et al [51] was used. The acute toxicity test was carried out for all the groups to evaluate any possible toxicity. When there is no information on acute toxicity testing of chemicals, the beginning dose of 300 mg/kg body weight is advised for animal welfare considerations [52]. Mice (*n* = 6) of either sex were treated with different doses (300, 400, 500, 600 mg/kg) [52–54] of the test compounds A and B, while the control group received DMSO (10 ml/kg,i.p). All the groups were observed for any gross effect for the first 4 h and then mortality was observed up to 2 weeks. Initial observations included nervousness, excitement, changes in body weight of mice before and after test compound administration, onset and signs of toxicity such as a change in skin and fur, eyes and mucous membrane changes, respiratory disturbances, circulatory changes, a sign of tremors, convulsions, lethargy, sleep and coma [52, 55].

##### Ethics statement animal experimentation

Adult male and female Wistar rats (200-300 g) and albino mice of mix gender (20-30 g) were used for the current study. The animals were kept in a controlled environment (23-25°c) at the animal house, Faculty of Pharmacy, University of Sargodha, and Sargodha. Animals were properly fed a standard diet and tap water. Animal handling was according to the National research guidelines [33]. Prior the animal experiment, our pharmacological protocols were approved by institutional ethical committee, College of Pharmacy, University of Sargodha, Pakistan with approval No (67B18 IAEC/UOS). All procedures used in this study were carried out in accordance with the ARRIVE guidelines. Animals were released after the experiments.

#### Statistical analysis

The results obtained were expressed as mean ± SEM (Standard error of mean) of six animals. Statistical analysis was done using Graph Pad prism demo software. One way analysis of variance (ANOVA) was also used followed by post-Dunnett’s test. Values were considered to be significant at the *P* < 0.05 level [56].

## Results

### Anti-inflammatory activity carrageenan-induced paw edema

**Fig. 2:**
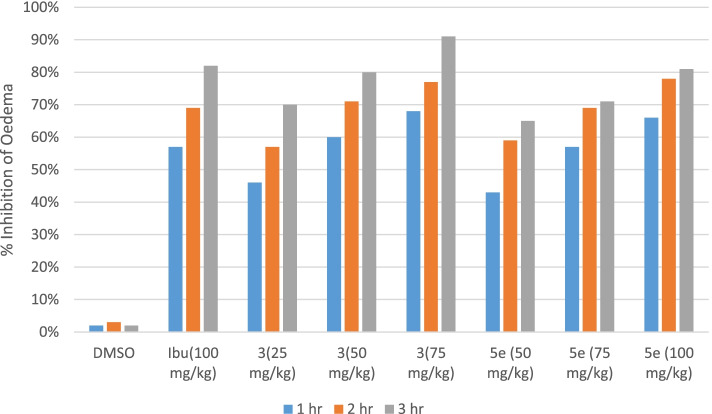
Comparison of anti-inflammatory effects of synthetic test compounds in carrageenan treatment groups of mice. Data expressed as mean ± SEM, one-way ANOVA followed by post-Dunnett’s test, *n* = 6

The results of significant and consistent anti-inflammatory activity of the test compounds are shown in Table 1, Fig. 2. There was a gradual increase in paw edema of mice in the control group from 1st hour till 3rd hour. The test compound **A** showed a highly significant (*P* < 0.001) decrease in paw edema ranging from 46 to 68% during the 1st hour of the experiment as compared to standard drug ibuprofen (57%). The second compound **B** showed 43 to 66% inhibition of edema during 1st hour. After 3 h maximum inhibition of edema was found for compound **A** (91%) as compared to reference drug ibuprofen (82%), while **B** showed equipotent results to ibuprofen (81%).

**Table 1:** Anti-inflammatory effect of test compound during Carrageenan- induced paw edema in mice

Groups	Treatments	Dose mg/kg	Paw edema in mm at different time intervals			% age inhibition of edema		
			1 h	2 h	3 h	1 h	2 h	3 h
1	DMSO	10 ml	1.3 ± 0.026	1.467 ± 0.056	1.6 ± 0.039	0	0	0
2	Ibuprofen	100	0.55 ± 0.043**	0.450 ± 0.043***	0.283 ± 0.031***	57%	69%	82%
3	**3**	25	0.7 ± 0.037*	0.633 ± 0.061**	0.483 ± 0.048***	46%	57%	70%
4		50	0.517 ± 0.048**	0.433 ± 0.049***	0.317 ± 0.031***	60%	71%	80%
5		75	0.417 ± 0.04***	0.33 ± 0.033***	0.15 ± 0.022***	68%	77%	91%
6	**5**	50	0.733 ± 0.049*	0.6 ± 0.037**	0.567 ± 0.056***	43%	59%	65%
7		75	0.55 ± 0.043**	0.45 ± 0.043***	0.467 ± 0.033***	57%	69%	71%
8		100	0.45 ± 0.043***	0.317 ± 0.031***	0.3 ± 0.037***	66%	78%	81%

Note. One way ANOVA as applied to compare the activity of control and test compound treated groups. (*n* = 6)

^*^significant inhibition of paw edema (*P* < 0.05)

^**^Very significant inhibition of paw edema (*P* < 0.01)

^***^Highly significant inhibition of paw edema (*P* < 0.001)

#### Egg-albumin induced paw edema in mice

**Fig. 3:**
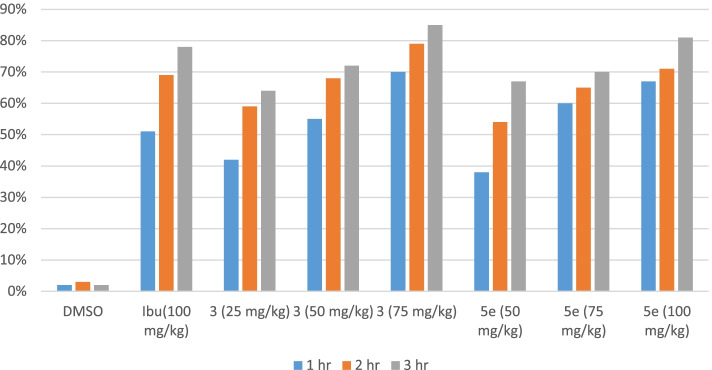
Comparison of anti-inflammatory effects of synthetic test compounds on egg-induced paw oedema in mice. Data expressed as mean ± SEM, one-way ANOVA followed by post-Dennett’s test, *n* = 6

Results have revealed that after 1 h of egg albumin administration test compound **A** at 75 mg/kg and **B** at 100 mg/kg exerted highly significant (*P* < 0.001) inhibition of paw edema in mice as compared to the control group and ibuprofen treated group (*P* < 0.01). After 3 h maximum inhibition of edema was found for compound **A** (85%) as compared to reference drug ibuprofen (78%), while **B** showed an 81% reduction in edema. All data related to egg induced paw edema is shown in Table 2, Fig. 3.

**Table 2:** Anti-inflammatory effect of test compounds in egg albumin- induced paw edema

Groups	Treatments	Dose mg/kg	Paw edema at different time intervals			%age inhibition of edema		
			1 h	2 h	3 h	1 h	2 h	3 h
1	DMSO	10 ml/kg	1.267 ± 0.02	1.417 ± 0.031	1.640 ± 0.040	0	0	0
2	Ibuprofen	100	0.617 ± 0.048**	0.433 ± 0.067***	0.367 ± 0.033***	51%	69%	78%
3	**3**	25	0.733 ± 0.067*	0.583 ± 0.07**	0.583 ± 0.070***	42%	59%	64%
4		50	0.567 ± 0.049**	0.45 ± 0.043***	0.45 ± 0.050***	55%	68%	72%
5		75	0.383 ± 0.031***	0.3 ± 0.037***	0.250 ± 0.022***	70%	79%	85%
6	**5**	50	0.783 ± 0.03*	0.650 ± 0.076**	0.533 ± 0.056***	38%	54%	67%
7		75	0.5 ± 0.04**	0.5 ± 0.037***	0.483 ± 0.054***	60%	65%	70%
8		100	0.417 ± 0.065***	0.417 ± 0.031***	0.317 ± 0.031***	67%	71%	81%

Note. One way ANOVA as applied to compare the activity of control and test compound treated groups. (*n* = 6)

^*^significant inhibition of paw edema (*P* < 0.05)

^**^Very significant inhibition of paw edema (*P* < 0.01)

^***^highly significant inhibition of paw edema (P < 0.001)

### Acetic acid writhing test

**Fig. 4:**
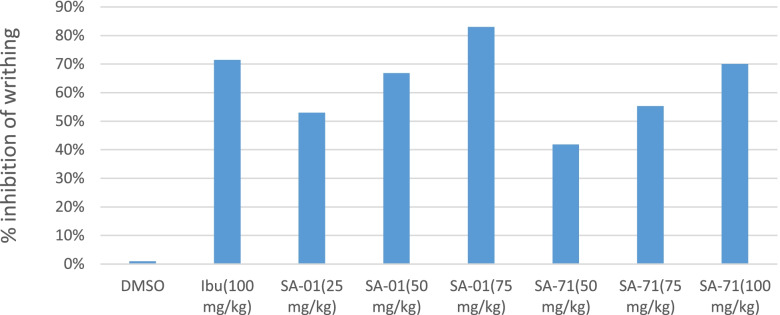
% age inhibition of acetic acid induced writh in mice showing analgesic activity of test compounds. Data expressed as mean ± SEM, one-way ANOVA followed by post-Dunnett’s test, *n* = 6

The administration of a 10 ml/kg dose of 3% acetic acid I.P produced a substantial number of wriths in the control mice pretreated with 10 ml/kg dose I.P of 5% DMSO solution. The mean percentage % inhibition of number of wriths of test compound A at doses 25, 50, 75 mg/kg were calculated to be 53, 66.83 and 83% respectively. The 75 mg/kg dose group showed maximum anti-nociceptive action, even more than the standard drug ibuprofen (100 mg/kg) treated group with % age inhibition of 71%. Similarly, the test compound B reduced the number of wriths elicited by acetic acid. The 100 mg/kg dose of B showed highly significant (*P* < 0.001) inhibition of writhing and its effect was comparable with the ibuprofen (71.5%) treated group. All data related to acetic acid-induced wriths have been explained in Table 3, Fig. 4.

**Table 3:** Data of Acetic acid-induced wriths in mice

Treatments	Doses mg/kg (i.p)	No of wriths	% age inhibition
3	25	38.33 ± 2.679**	53%
	50	27.66 ± 1.80***	66.83%
	75	14.0 ± 0.856***	83%
5	50	47.66 ± 2.90*	41.83%
	75	36.66 ± 1.978**	55.30%
	100	24.83 ± 1.759***	70%
Ibuprofen	100	23.5 ± 1.05***	71.5%
DMSO(10 ml/kg)		83.33 ± 3.37	0

% age inhibition of wriths in mice in all groups showing analgesic activity of synthetic test compounds. Data expressed as mean ± SEM, one-way ANOVA followed by post Dunnett’s test, *n* = 6

^*^Significant inhibition of nociceptive response (*P* < 0.05)

^**^very significant inhibition of nociceptive response (*P* < 0.01)

^***^Highly significant inhibition of nociceptive response (*P* < 0.001)

### Tail immersion

In the tail immersion model of thermal pain, the test compounds increased the pain threshold significantly during the period of observation and indicated the participation of a higher centre. It was observed that ibuprofen showed a significant analgesic effect by increasing the pain threshold at 0, 15, 30 and 60 min. Highly significant analgesia was observed at 60 min where the pain threshold was highly increased by ibuprofen (13.45 ± 0.287*) as compared to the control DMSO (3.367 ± 0.145).

**Fig. 5:**
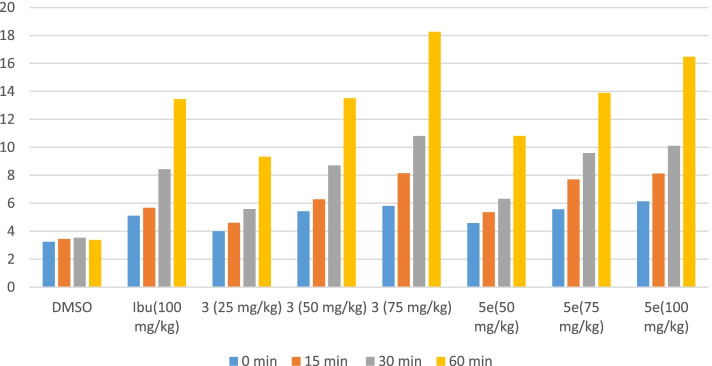
Analgesic activity results of the tail-flick experiment in mice. Data expressed as mean ± SEM, one-way ANOVA followed by post-Dunnett’s test, *n* = 6

At 30 and 60 min, time interval compound **A** at 50 mg/kg dose showed highly significant inhibition (*P* < 0.001) of pain. Peak response was observed with **A** at 75 mg/kg dose showing highly significant inhibition of pain by increasing the pain threshold (18.267 ± 0.217*) as compared to the control group (3.367 ± 0.145). The second test compound **B** also showed a significant increase in the pain threshold at different time intervals. **B** at 75 mg/kg dose exhibited a highly significant reduction in pain response at 0, 15, 30 and 60 min. Interestingly by increasing the dose of **B** to 100 mg/kg also increased the pain threshold at all times intervals with a peak response at 60 min (16.48 ± 0.288*) as compared to the control group. The result of test compounds **A** (50 mg/kg) and **B** (75 mg/kg) at 60 min was comparable with the standard drug ibuprofen (100 mg/kg) at the same time interval Table 4, Fig. 5.

**Table 4:** Analgesic effects of synthetic test compounds in thermal-induced nociception model

Groups	Treatments	Dose mg/kg	Minutes after treatment			
			0	15	30	60
Response in sec (Mean ± SEM)						
1	**DMSO**	10 ml/kg	3.238 ± 0.125	3.45 ± 0.056	3.53 ± 0.076	3.367 ± 0.145
2	**Ibuprofen**	100	5.1 ± 0.068**	5.667 ± 0.136**	8.43 ± 0.123***	13.45 ± 0.287***
3	**3**	25	4.0 ± 0.082*	4.6 ± 0.089*	5.58 ± 0.119**	9.33 ± 0.133**
4		50	5.417 ± 0.111**	6.28 ± 0.095 **	8.71 ± 0.207***	13.517 ± 0.250***
5		75	5.8 ± 0.1***	8.15 ± 0.177 ***	10.81 ± 0.119***	18.267 ± 0.217***
6	**5**	50	4.583 ± 0.149*	5.36 ± 0.133**	6.317 ± 0.079**	10.817 ± 0.199**
7		75	5.567 ± 0.138**	7.7 ± 0.142***	9.583 ± 0.087***	13.9 ± 0.146***
8		100	6.13 ± 0.165***	8.121 ± 0.185***	10.117 ± 0.149***	16.48 ± 0.288***

Analgesic effect of compounds, Data expressed as mean ± SEM, one-way ANOVA followed by post Dunnett’s test, (*n* = 6)

^*^Significant inhibition of nociceptive response (*P* < 0.05)

^**^very significant inhibition of nociceptive response (*P* < 0.01)

^***^Highly significant inhibition of nociceptive response (*P* < 0.001)

#### Formalin-induced nociception

Formalin administration generated a typical pattern of flinching and licking behaviour in mice. The test compounds exerted a significant dose-dependent inhibitory effect on both phases of the formalin test. The results (Table. 5) showed that the time spent licking the paw was significantly (*P* < 0.05) reduced by **A** (25 mg/kg). The % age inhibition of licking in Group II treated with ibuprofen was found to be 19%. It was interesting to note that all the doses of synthetic compounds and standard drug ibuprofen showed profound effects in the late phase of nociception.

**Table 5:** Analgesic effect of synthetic test compounds during formalin-induced nociception in mice

Groups	Dose (mg/kg)	Licking time (sec)		% age Inhibition	
		1st Phase	2nd Phase	1st Phase	2nd Phase
3	25	128.8 ± 3.609*	572.5 ± 4.603*	18%	25%
	50	108 ± 5.538**	291.1 ± 2.482***	31%	62%
	75	94.83 ± 2.91**	187.6 ± 3.756***	39%	75%
5	50	135.1 ± 4.262*	545.8 ± 10.59*	14%	28%
	75	125.3 ± 3.71*	394.83 ± 12.3**	20%	48%
	100	100.6 ± 5.998**	287 ± 2.236***	36%	62%
Ibuprofen	100	126.5 ± 4.137*	274.8 ± 3.02***	19%	64%
DMSO(10 ml/kg)		156.83 ± 4.46	761.1 ± 13.7	0	0

%age inhibition of nociceptive response in mice, Data expressed as mean ± SEM, one-way ANOVA followed by post Dunnett’s test, (*n* = 6)

^*^Significant inhibition of nociceptive response (*P* < 0.05)

^**^Very significant inhibition of nociceptive response (*P* < 0.01)

^***^Highly significant inhibition of nociceptive response (*P* < 0.001)

**Fig. 6:**
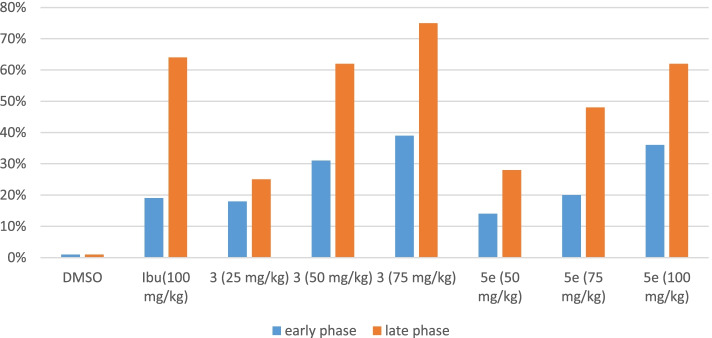
% age inhibition of pain in mice treated with different doses of test compounds formalin-induced paw licking. Values = Mean ± SEM

In the late phase, the % age inhibition of licking in the standard group was 64% which was comparable to compound **A** (62%) at 50 mg/kg dose. Similarly, **B** at 100 mg/kg dose also produced 62% licking inhibition, whereas **A** at 75 mg/kg dose showed more profound inhibition of licking (75%). This group showed maximum analgesic effects among all the treated groups. Results in Table 5, Fig. 6.

#### Brewer’s yeast-induced pyrexia

**Fig. 7:**
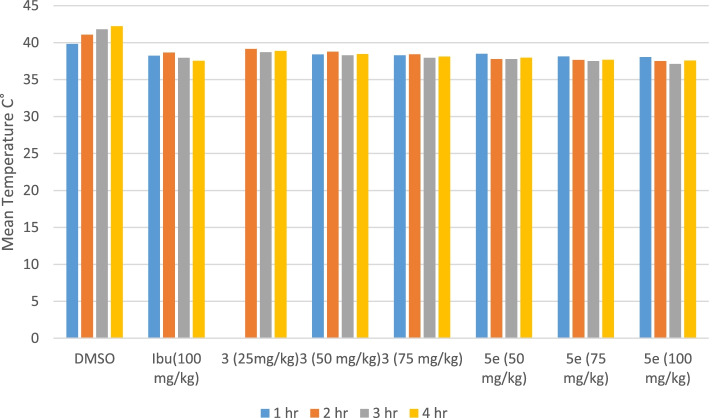
Effect of synthetic test compounds on yeast-induced hyperpyrexia in rats (mean ± SEM)

The anti-pyretic activity of test compounds was evaluated by inducing fever in rats. Following administration of brewer’s yeast in all groups of rats, the rectal temperature of rats after 18 h was increased by 0.7 °C. The mean temperature of the control group rats was 39.83 °C, 41.06 °C, 41.78 °C and 42.23 °C after 1, 2, 3 and 4 h, respectively. Ibuprofen at 100 mg/kg dose, G4 and G5 (compound A treated groups) exhibited a highly significant (*P* < 0.001) reduction of rectal temperature in rats after the 3rd and 4th hour. Rats in G6, G7 and G8 (compound B treated) showed even better antipyretic activity by showing highly significant (*P* < 0.001) reduction in pyrexia after the 2nd,3rd and 4th hour as compared to the negative control group, Table 6, Fig. 7.

**Table 6:** Effect of test compounds during yeast induced hyperpyrexia in rats

Treatment	Groups	Dose mg/kg	Basal Temperature(°C)	Rectal Temperature (°C) 18 h after yeast induced pyrexia	Rectal temperature (°C) after treatment with extract			
					1 h	2 h	3 h	4 h
DMSO	**G1**	(10 ml/kg)	38.07 ± 0.22	38.8 ± 0.29	39.83 ± 0.105	41.067 ± 0.301	41.78 ± 0.22	42.23 ± 0.15
Ibuprofen	**G2**	100	37.4 ± 0.20	38.3 ± 0.20	38.23 ± 0.084**	38.650 ± 0.123**	37.93 ± 0.191***	37.53 ± 0.105***
3	**G3**	25	38.58 ± 0.24	39.8 ± 0.10	39.13 ± 0.092*	39.15 ± 0.089**	38.71 ± 0.083**	38.87 ± 0.084**
	**G4**	50	38.6 ± 0.083	39.3 ± 0.059	38.41 ± 0.105**	38.76 ± 0.115**	38.283 ± 0.122***	38.46 ± 0.080**
	**G5**	75	37.8 ± 0.28	38.9 ± 0.084	38.28 ± 0.083**	38.43 ± 0.154**	37.93 ± 0.115***	38.1 ± 0.124***
5	**G6**	50	37.7 ± 0.26	39.6 ± 0.09	38.483 ± 0.07**	37.78 ± 0.224***	37.76 ± 0.249***	37.98 ± 0.185***
	**G7**	75	37.6 ± 0.161	38.4 ± 0.08	38.150 ± 0.106**	37.65 ± 0.161***	37.5 ± 0.14***	37.68 ± 0.117***
	**G8**	100	37.4 ± 0.22	38.6 ± 0.121	38.067 ± 0.076**	37.5 ± 0.161***	37.1 ± 0.103***	37.567 ± 0.161***

*G* Group

^*^Significant inhibition of pyrexia (*P* < 0.05)

^**^Very Significant inhibition of pyrexia (*P* < 0.01)

^***^Highly Significant inhibition of pyrexia (*P* < 0.001)

## Discussion

The present study was designed to evaluate the in vivo anti-inflammatory, analgesic, and antipyretic activities of newly synthesized compounds A and B which are triazole derivatives with a history of significant pharmacological activities. We hypothesized that these compounds A and B can be a potential therapeutic candidate for different ailments like inflammation, analgesia, and pyrexia. The present study came up with some novel findings. Both the compounds were found effective therapeutic agents as anti-inflammatory, analgesic, and antipyretic [57].

The most widely used test for the screening of new anti-inflammatory agents is the carrageenan-induced edema in the rat/mice hind paw [58]. Carrageenan-induced paw edema is considered as a model of an acute inflammatory process involving several mediators release in a sequence. Doses to be used in this study were evaluated by the pharmacologically established procedures [36]. The injection of carrageenan and egg albumin in mice paw initiates a biphasic release of inflammatory mediators. Other events occurring during paw edema include increased tissue water and plasma proteins exudation along with neutrophils extravasation [59]. Paw edema is mediated by the initial release of serotonin, bradykinin and histamine, during the 1-h post carrageenan injection. This is followed by the release of prostaglandins, protease and lysosome at around the 3rd hour and lasts for about 6 h after carrageenan injection [60]. In the current study, the I.P administration of both test compounds A in doses (75 mg/kg) and B (100 mg/kg) showed a highly significant (*P* < 0.001) decrease in paw edema during the 1st hour of the experiment as compared to standard drug ibuprofen. After 3 h. Compound A showed maximum inhibition of edema (91%) then reference, while **B** showed equipotent results to ibuprofen (81%) as shown in Table 1. We can speculate on basis of the hind paw model of inflammation that compounds A and B may inhibit these mediators in the early stage of acute inflammation [36, 59, 61].

.The reduction of oedemogenesis in the early phase of inflammation was shown by both compounds **A** and **B** suggesting their effectiveness in inhibiting the release or the action of early phase mediators that reach the insulted site immediately thereby decreasing the vascular permeability, fluid exudation and thus fairly suppress the edema. Both of the compounds also showed significant anti-inflammatory activity in the second phase as well, which is thought to occur due to suppression of prostaglandins, kinins and proteases synthesis induced by carrageenan and egg albumin during this phase [62].

Both test compounds also showed highly significant (*P* < 0.001) inhibition of paw edema in mice as compared to the control group and ibuprofen treated group (*P* < 0.01). after 3 h both compounds showed higher inhibition of edema as compared to reference drug ibuprofen (78%). results demonstrated that test compounds exert anti-inflammatory effects by possibly blocking the release of 5-hydroxytryptamine (5-HT) and histamine, the inflammatory mediators released by egg albumin.

The formalin and acetic acid tests (chemical stimuli) have been used to elucidate the central and peripheral mediated pain, while the tail immersion test (thermal stimuli) was applied for the assessment of centrally mediated pain [63]. In the current study, the standard drug ibuprofen and test compounds modulated both the early and late phases of pain induced, and its anti-nociceptive effect was dose-dependent and more pronounced in the late phase. The results have suggested that the analgesic activity of test compounds could be due to their central action as proposed by Amanlou et al. [58]

The acetic acid-induced writhing is a chemically induced visceral pain model that has been associated with the release of cyclooxygenase, bradykinin, arachidonic acid, histamine and substance P, exciting pain nerve endings leading to abdominal writhing [64].In this study, the reference drug ibuprofen and test compounds A and B showed a significant reduction (*P* < 0.001) in the number of wriths as compared to the control group. It may be deduced that test compounds have reduced the number of wriths by inhibiting the release of one of these mediators of the pain.

The writhing caused by acetic acid in mice was significantly reduced by our 1, 2, 4 Triazole derivatives. This proposed that part of the analgesic effect may be peripherally mediated like NSAIDs, however the derivatives also showed significant anti-nociceptive effect in tail immersion test. The tail flick and tail immersion tests are acute thermic and phasic pain models that have been shown to be selectively suppressed by opioid-like analgesic drugs that act centrally [65]. As a result, it’s conceivable that our compounds have NSAID-like, as well as opioid-like analgesic action (central and peripheral analgesic activities).

Yeast-induced fever is called pathogenic fever and it has been well documented as the most economical and reliable method [50]. The presence of proteins in yeast is linked to fever via inflammatory reaction in this method [10]. Test compounds A and B have shown a reduction in yeast induced fever in the present study as shown in Table 6. Test compound **A** at 75 mg/kg dose and **B** at 50, 75 and 100 mg/kg doses showed a highly significant (*P* < 0.001) decrease in fever. Maximum reduction in fever was observed with test compound **B** at 100 mg/kg dose, its response was even potent than the ibuprofen treated group. It has been well established that the prostaglandin E_2_ (PGE_2_) is the final fever mediator in the brain, especially in the preoptic area of the anterior hypothalamus [66]. Thus it may be suggested that the test compounds inhibited the biosynthesis of prostaglandins through their inhibitory action on Cyclooxygenase COX enzymes.

## Conclusion

In nutshell, compounds **A** and **B** are a potential candidate for anti-inflammatory, analgesic and anti-pyretic activities. However, the mechanism of each activity should be explored to unfold the pathway for anti-inflammatory, analgesic, and anti-pyretic activities. Our research would definitely provide help for pharmaceutical researchers to conduct more organized and fertile drug discoveries using this scaffold with modifications to enhance pharmacokinetic profile and resolving issues related to side effects of traditional analgesic anti-inflammatory drugs.

## Data Availability

The datasets used and/or analyzed during the current study are available from the first author Tabinda Azim (tabinda.azim@iqraisb.edu.pk) on reasonable request.

## Abbreviations

PGE_2_: Prostaglandin E_2_
COX enzymes: Cyclooxygenase (COX)
5 HT: 5-hydroxytryptamine
IR: Infrared
NMR: Nuclear magnetic resonance
TNF-α: Tumor necrosis factor-α
NRC: National research council
